# Cyclooxygenase-1 as the Main Source of Proinflammatory Factors After Sodium Orthovanadate Treatment

**DOI:** 10.1007/s12011-014-0176-4

**Published:** 2014-11-15

**Authors:** Jan Korbecki, Irena Baranowska-Bosiacka, Izabela Gutowska, Katarzyna Piotrowska, Dariusz Chlubek

**Affiliations:** 1 Department of Biochemistry and Medical Chemistry, Pomeranian Medical University, Powstańców Wlkp. 72 Av., 70-111 Szczecin, Poland; 2 Department of Biochemistry and Human Nutrition, Pomeranian Medical University, Broniewskiego 24 Str., 71-460 Szczecin, Poland; 3 Department of Physiology, Pomeranian Medical University, Powstańców Wlkp. 72 Av., 70-111 Szczecin, Poland

**Keywords:** Prostaglandin, Cyclooxygenase, Macrophage, Vanadium, Sodium orthovanadate

## Abstract

Vanadium is a metal present in air pollution. Its compounds may have both anticancer and carcinogenic properties. Vanadium compounds are tested in treatment of diabetes and cancer. An important research direction aimed at better understanding of the mechanisms of action of the vanadium compounds is a more detailed insight into their impact on inflammatory reactions. The aim of this study was to examine the effect of micromolar concentrations of sodium orthovanadate, Na_3_VO_4_, on the activity and expression of cyclooxygenases: COX-1 and COX-2. PMA-activated THP-1 macrophages were incubated in vitro for 48 h with micromolar concentrations of sodium orthovanadate. As shown by an ELISA assay, sodium orthovanadate increases the quantity of prostaglandin E_2_ being released into the medium in a dose-dependent manner as well as impacts the quantity of the stable metabolite of thromboxane A_2_: thromboxane B_2_. The use of a COX-2 inhibitor, NS-398, revealed that this effect was independent of changes in the activity of COX-2. Western blotting analysis showed that sodium orthovanadate increased the expression of COX-2 when used with NS-398. Quantitative real-time PCR measurements of mRNA levels of genes *PTGS1* and *PTGS2* revealed no effect of the tested vanadium compound on the levels of analyzed transcripts.

## Introduction

Vanadium is a metal present in the polluted air in large urban and industrial agglomerations. Environmental sources of vanadium include soil and pollution from combustion of fossil fuels [1]. Prolonged exposure to vanadium oxide contained in the air may cause lung cancers [2]. Also, other inorganic vanadium compounds are known to have carcinogenic properties. The mechanism of action of these compounds has been well studied and proceeds along multiple pathways. Vanadium compounds stimulate proliferation of cells while simultaneously interfering with the course of apoptosis [3]. However, in vitro studies also revealed that the treatment of cancer cells with vanadium compounds caused apoptosis and inhibition of proliferation [4, 5]. Depending on the expression of p53, vanadium compounds have either carcinogenic or anticancer properties [3].

Molecular mechanisms of action of vanadium compounds are still being studied. Thanks to the easily occurring changes in their oxidation states, vanadium compounds generate reactive oxygen species (ROS) by means of Fenton’s reaction or a reaction with oxygen [6]. In addition, compounds in question are structurally similar to phosphate anions. Thus, they may act as protein tyrosine phosphatase (PTP) inhibitors [7]. As a result of inhibition of these enzymes, numerous signaling pathways are activated within cells.

Vanadium compounds are tested in experimental treatment of diabetes as they inhibit PTP1B, among their other effects [8]. In type 2 diabetes, the activity of this phosphatase is increased, leading to insulin resistance. In addition, vanadium compounds enhance the glucose transport mediated by glucose transporter type 4 (GLUT-4) and the glycogen synthesis, leading to reduced blood glucose levels [9]. However, due to the higher toxicity of inorganic vanadium compounds, organic vanadium derivatives such as bis(maltolato)oxovanadium(IV) (BMOV) are being tested for their potential use in antidiabetic treatment [10].

An important research direction aimed at better understanding of the mechanisms of action of the vanadium compounds is a more detailed insight into their impact on inflammatory reactions. These reactions are associated with numerous disorders. They are important in the course of cancers or atherosclerosis. One of the most important inflammatory processes is the synthesis of prostaglandins and thromboxanes, occurring along cyclooxygenase pathway from arachidonic acid released from membrane phospholipids by means of cytosolic phospholipase A_2_ (cPLA_2_). In physiological conditions, cyclooxygenase-1 (COX-1) plays an important role in the synthesis of prostaglandin E_2_ (PGE_2_). The expression of this enzyme is maintained at the constitutive level and is rarely changed. Expression of cyclooxygenase-2 (COX-2) is increased in inflammatory reactions. Increased enzyme levels lead to increased production of prostaglandins.

Increased expression of COX-2 in tumor cells is very important for the natural history of the disease. PGE_2_ being synthesized by this enzyme disturbs apoptosis and promotes angiogenesis, thus promoting tumor growth [11]. Potential anticancer drugs should be examined for their effects on expression and activity of COX-2 and induction of inflammatory reactions, as this would allow for development of safe therapies.

Considering the above, the aim of this study was to demonstrate the effect of micromolar concentrations of sodium orthovanadate on the development of inflammation in THP-1 macrophage cells by measuring the activity and expression of COX-1 and COX-2 (at both mRNA and protein levels).

## Material and Methods

### Cell Culture

The study was conducted on macrophages obtained from a monocytic line THP-1. Human THP-1 monocytic cells have been widely employed as an in vitro model for investigating the molecular mechanisms underlying monocyte-to-macrophage differentiation [12]. THP-1 cells (ATCC, Rockville, USA) were differentiated into macrophages by administering phorbol myristate acetate (PMA) [12, 13] and cultured in RPMI-1640 medium (BIOMED-LUBIN, Poland) supplemented with 10 % FBS without fatty acid (ALAB, Poland), penicillin (100 U/mL), and streptomycin (100 mg/mL; Sigma-Aldrich, Poland) at 37 °C in 5 % CO_2_. The THP-1 monocytes were treated with 100 nM PMA (Sigma-Aldrich, Poland) for 24 h, and then the adherent macrophages were washed three times with PBS (BIOMED-LUBLIN, Poland) and incubated with sodium orthovanadate (Na_3_VO_4_) (Sigma-Aldrich, Poland) solution with or without specific COX-2 inhibitor NS-398 (Sigma-Aldrich, Poland) at final concentration of 50 μM for 48 h at 37 °C. Incubation time was selected on the basis of results obtained in preliminary experiments. Sodium orthovanadate was used at final concentrations of 80 nM, 1, 4, and 10 μM. These concentrations were selected on the basis of in vitro study of cancer cells [5] and study on blood vanadium concentration in Taiwanese students [14]. After incubation, cells were harvested by scraping and a pellet was obtained by centrifugation (250*g* for 5 min).

The cell count was determined with a Bright Line Hemacytometer (Sigma-Aldrich, Poznan, Poland). Cell viability was examined using a trypan blue dye exclusion method. Cell cultures with viability more than 97 % were used for experiments [15]. Protein concentration was measured by the Bradford method [16].

### Measurements of COX-1 and COX-2 Activity

Cyclooxygenases COX-1 and COX-2 activity were measured in vitro by quantitative measurement of their products: PGE_2_ and thromboxane A_2_ (TXA_2_). The cells were incubated for 48 h with sodium orthovanadate solutions, as described above. PGE_2_ and TXA_2_ were extracted from the culture supernatants with the use of Bakerbond SPE columns (J.T. Baker, USA), as described in manufacturer’s instructions. The concentrations of PGE_2_ released were measured spectrophotometrically by using the PGE_2_ enzyme immunoassay kit (Cayman Chemical, USA) according to the manufacturer’s protocol. As TXA_2_ has a short half-life (37 s) and is rapidly hydrolyzed nonenzymatically to its stable derivative thromboxane B_2_ (TXB_2_), the thromboxane B2 enzyme immunoassay kit (Cayman Chemical, USA) was used to measure free TXA_2_ indirectly.

### Western Blotting Analysis of COX-1 and COX-2 Expression

Cells after incubation with sodium orthovanadate were washed with PBS. After scraping, they were lysed with lysing buffer containing protease inhibitor, ethylene-diaminetetra-acetic acid 5 mM, sodium dichloroisocyanurate 1 %, TRITON-X 1 %, sodium orthovanadate 100 mM (Sigma-Aldrich, Poland), and equal amounts of protein were separated in gel electrophoresis and transferred to a nitrocellulose membrane (Thermo Scientific, Pierce Biotechnology, USA) at 157 mA for 2 h at room temperature. After blocking the membrane with 5 % non-fat milk in Tris-buffered saline (Sigma-Aldrich, Poland) containing 0.1 % Tween 20 (Sigma-Aldrich, Poland) for all night at 4 °C, it was incubated with primary monoclonal antibodies direct against COX-1 or COX-2 (Santa Cruz Biotechnology, USA) in dilution 1:200 with a monoclonal anti-ß-actin (1:5000; Santa Cruz Biotechnology, USA) and next with secondary antibodies (goat anti-mouse IgG HRP; Santa Cruz Biotechnology, USA) in dilution 1:5000. Signals were visualized by chemiluminescence (Thermo Scientific, Pierce Biotechnology, USA). ImageJ 1.41o (NIH, USA) was used to densitometric analysis of bands.

### Imaging of COX-1 and COX-2 Immunoexpression

Expression of COX-1 and COX-2 proteins was examined with confocal microscopy. THP-1 cells were grown on cover glasses in standard in vitro culture conditions. Further, cells were washed with PBS and fixed with 4 % buffered formalin for 15 min in room temperature. After the fixation and washing with PBS, cells were permeabilized with 0.5 % solution of Triton X-100 in PBS. After washing with fresh portion of PBS, cells were incubated with mouse anti-COX-1 or anti-COX-2 primary antibodies (Santa Cruz Biotechnology, USA) in dilution 1:50, in 4 °C, overnight, and then washed and incubated with secondary antibody: anti mouse IgG FITC conjugated (Sigma-Aldrich, Poland) in dilution 1:60 in antibody diluent (Dako, Poland), 30 min in room temperature, and after washing with PBS further with Hoechst 33258, 30 min, room temperature. The cells were examined under a confocal microscope (FV1000 confocal with IX81 inverted microscope, Olympus, Germany), three channel acquisition and sequential scanning was used for best resolution of signal from Hoechst 33258 and FITC fluorescence. Additionally, fluorescent images were merged with transition light images.

### Quantitative Real-Time PCR Analysis (qRT-PCR) of COX-1 and COX-2 mRNA

Quantitative mRNA expression of *PTGS1* (NCBI Reference Sequence: NM_000962) and *PTGS2* (NM_000963) genes was performed in a two-step reverse transcription PCR. Total RNA was extracted from cells using RiboPure kit (Life Technologies, USA). After determination of the quantity and quality of isolated RNA using a NanoDrop ND-1000 spectrophotometer (NanoDrop Technologies, USA), complementary DNA (cDNA) was prepared using RevertAid First Strand cDNA Synthesis kit (Fermentas, Thermo Scientiic, Lithuania). Quantitative real-time PCR was performed in 7500 Fast Real-Time PCR System (Applied Biosystems, USA), using pre-validated Taqman Gene Expression Assays TaqMan GE Master Mix (Applied Biosystems, USA) and 1.5 μl of cDNA for each reaction mix of 15 μl. Each sample was analyzed in two technical replicates, and mean *C*_T_ values were used for further analysis. Calculations were performed using the ΔΔCt relative quantification method, using 7500 Fast Real-Time PCR System Software (Applied Biosystems, USA). The thresholds were set manually to compare data between runs, and *C*_T_ values were extracted. All *C*_T_ values for each sample were normalized to the value obtained for *GAPDH* and *HPRT1*, the endogenous control gene. Fold change between groups was calculated from the means of the logarithmic expression values.

### Statistical Analysis

Arithmetical means and the standard deviations (±SD) were calculated for each of the studied quantitative parameters. The distribution of results for individual variables was obtained by Shapiro-Wilk *W* test. As most of the distributions deviated from normal distribution, non-parametric tests were used for further analyses. To assess the differences between the used concentration of vanadium, non-parametric Wilcoxon matched pairs test and Friedmann’s ANOVA were used. The obtained results were analyzed statistically using Statistica 10 (StatSoft, Poland) software, and *p* values of less than 0.05 were considered as significant.

## Results

### COX-1 is the Main Source of Proinflammatory Factors After Sodium Orthovanadate Treatment

**Fig. 1:**
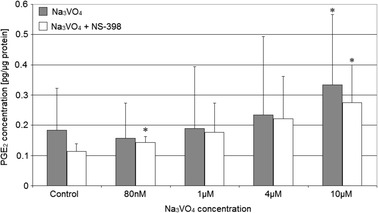
The quantity of PGE_2_ depending on sodium orthovanadate and NS-398. THP-1 macrophages were cultured with sodium orthovanadate solutions for 48 h. After incubation, cells were scrapped and PGE_2_ concentration was measured by using spectrophotometric method (*n* = 5). *p < 0.05, statistically significant as compared with control (Wilcoxon test)

Sodium orthovanadate added to the macrophages increased PGE_2_ synthesis in a dose-dependent manner; however, the significant results were obtained only for 10 μM vanadium solution (Fig. 1).

**Fig. 2:**
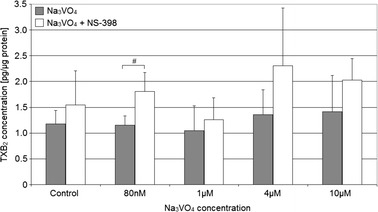
The quantity of TXB_2_ depending on sodium orthovanadate and NS-398. THP-1 macrophages were cultured with sodium orthovanadate solutions for 48 h. After incubation, cells were scrapped and TXB_2_ concentration was measured by using spectrophotometric method (*n* = 5). #*p* < 0.05, statistically significant as compared with the experiment with NS-398 (Mann-Whitney *U* test)

Similar results were obtained for measurement of TXB_2_ concentration in cell cultures depending on the cultivation conditions, but in such case, sodium orthovanadate addition to the cells cause no changes in TXA_2_ synthesis (Fig. 2).

The addition of NS-398—the selective COX-2 inhibitor to the macrophage culture with sodium orthovanadate caused a reduction of PGE_2_ and increase of TXB_2_ concentration vs. cultures without inhibitor, but there were no significant differences against TXB_2_ result for 80 nM sodium orthovanadate (*p* = 0.02). Additionally, the increase in sodium orthovanadate concentration was followed by an increase in PGE_2_ synthesis (for 10 μM sodium orthovanadate *p* = 0.04 compared to control) and TXA_2_ synthesis in macrophages.

The results show that orthovanadate at concentrations used increases the quantities of PGE_2_ being released by macrophage cells of the THP-1 line, but the process is independent on the activity of COX-2.

### Protein Expression of COX-1 Enzyme Decreased but COX-2 Enzyme Not Changed After Sodium Orthovanadate Addition to the Cells

**Fig. 3:**
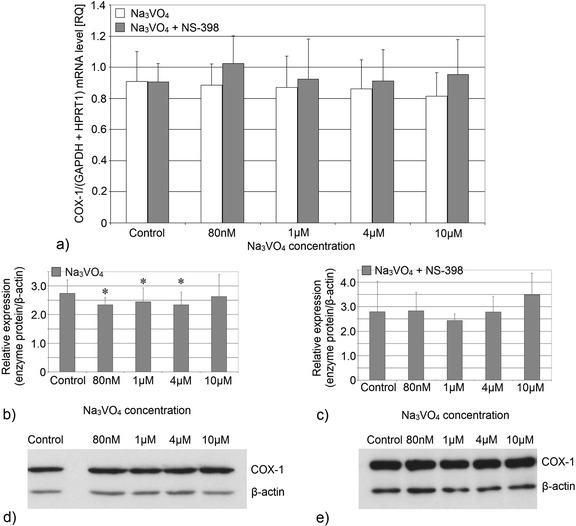
Effect of sodium orthovanadate on COX-1 protein and COX-1 mRNA expression. Effect of vanadium solutions on **a** COX-1 mRNA expression and **b** COX-1 protein expression (densitometric analysis of protein normalized to β-actin); **d** representative Western blot in macrophages cultured with sodium orthovanadate without and with NS-398 (**c** and **e**). THP-1 macrophages were cultured with sodium orthovanadate solutions for 48 h. After incubation, cells were harvested by scraping and mRNA was measured by using real-time PCR method (*n* = 3) and protein expression by using Western blotting method (*n* = 3). * statistically significant as compared with controls (Wilcoxon test)

**Fig. 4:**
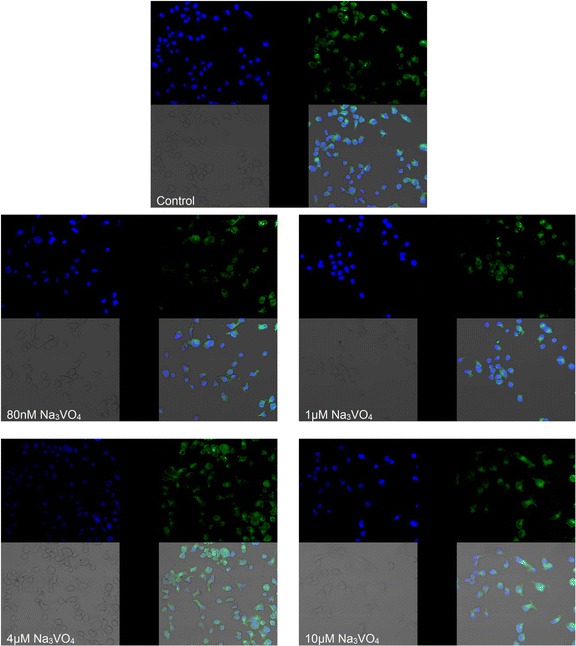
Imaging of COX-1 enzyme by fluorescence microscopy in macrophages cultured with sodium orthovanadate. THP-1 macrophages were cultured with sodium orthovanadate solutions for 48 h. The immunohistochemistry was performed using specific primary antibody, mouse anti-COX-1 (the overnight incubation at 4 °C), and secondary antibodies conjugated with fluorochrome - anti-mouse IgG-FITC (incubation for 45 min at room temperature). The nuclei of cells were DAPI stained. Image analysis was performed with a fluorescent microscope using filters 38 HE GFP for green fluorescence and 49 DAPI for blue fluorescence

Sodium orthovanadate lead to a 10–15 % reduction in COX-1 protein expression in macrophages cultured without NS-398 (Figs. 3 and 4). Statistically significant differences compared to control were observed for sodium orthovanadate at concentrations of 80 nM (*p* = 0.012), 1 μM (*p* = 0.04), and 4 μM (*p* = 0.012).

Addition of the NS-398 inhibitor and different concentrations of sodium orthovanadate into the culture had no effect on the expression of COX-1 protein in macrophages; however, a growing trend could be observed in COX-1 protein expression starting from sodium orthovanadate concentration of 4 μM.

**Fig. 5:**
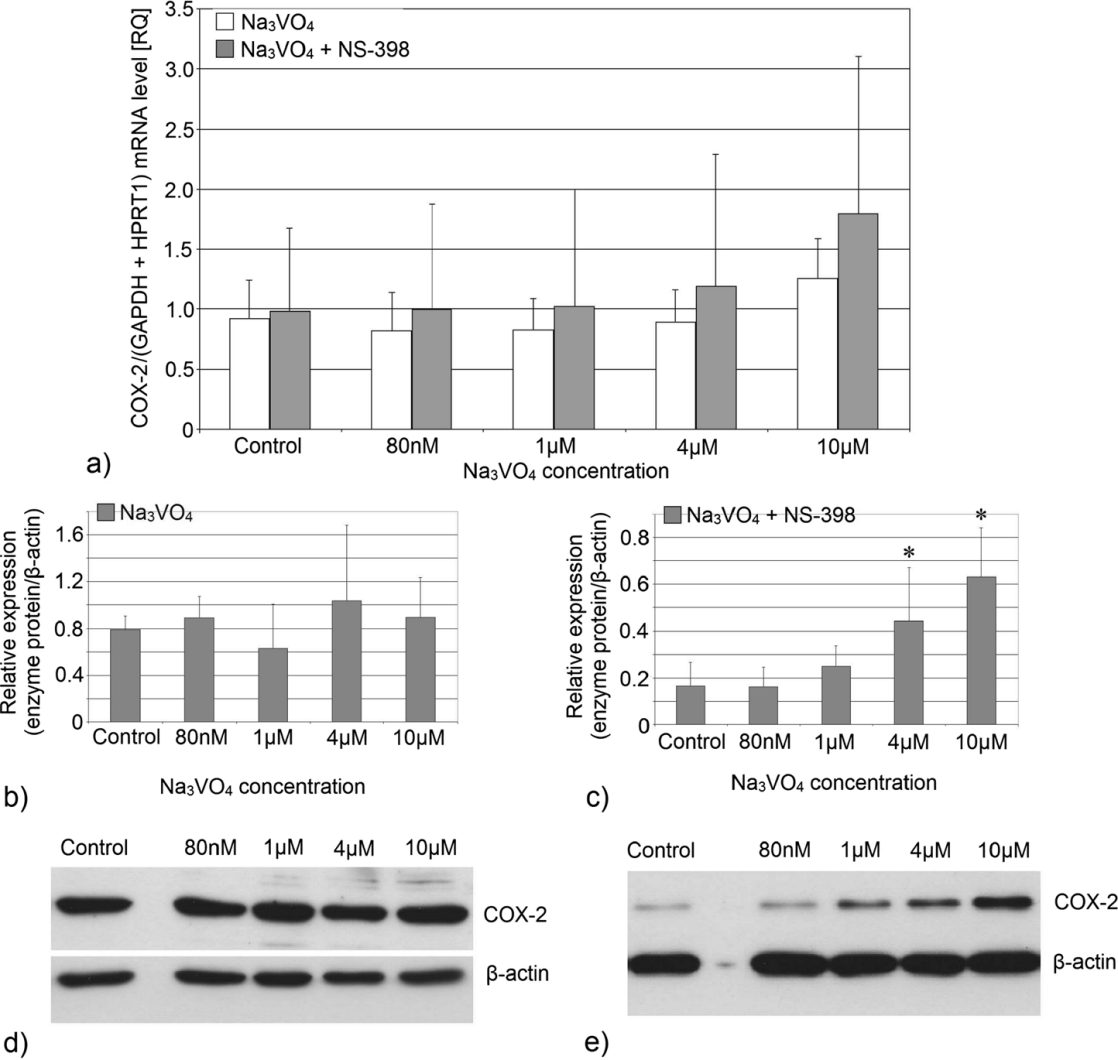
Effect of sodium orthovanadate on COX-2 protein and COX-2 mRNA expression. Effect of vanadium solutions on **a** COX-2 mRNA expression and **b** COX-2 protein expression (densitometric analysis of protein normalized to β-actin); **d** representative Western blot in macrophages cultured with sodium orthovanadate without and with NS-398 (**c** and **e**). THP-1 macrophages were cultured with sodium orthovanadate solutions for 48 h. After incubation, cells were harvested by scraping, and mRNA was measured by using real-time PCR method (*n* = 3) and protein expression by using Western blotting method (*n* = 3). * statistically significant as compared with controls (Wilcoxon test)

**Fig. 6:**
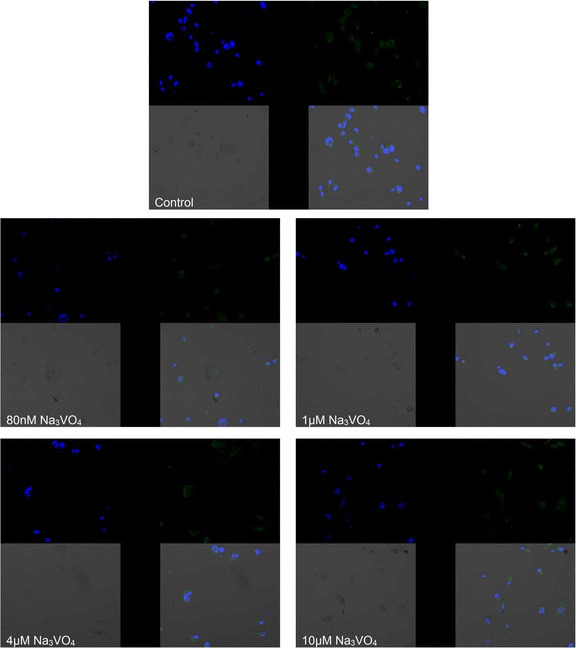
Imaging of COX-2 enzyme by fluorescence microscopy in macrophages cultured with sodium orthovanadate. THP-1 macrophages were cultured with sodium orthovanadate solutions for 48 h. The immunohistochemistry was performed using specific primary antibody, mouse anti-COX-2 (the overnight incubation at 4 °C) and secondary antibodies conjugated with flouorochrome - anti-mouse IgG-FITC (incubation for 45 min at room temperature). The nuclei of cells were DAPI stained. Image analysis was performed with a fluorescent microscope using filters 38 HE GFP for green fluorescence and 49 DAPI for blue fluorescence

The studied vanadium compound had no significant effect on the expression of COX-2 protein in THP-1 cells when used at any concentration; however, the addition of COX-2 inhibitor lead to a significant increase in expression that was dependent on concentration of the vanadium compound (Figs. 5 and 6). Statistically significant differences were observed for sodium orthovanadate solutions at concentrations of 4 μM (*p* = 0.03; 250 % increase compared to control) and 10 μM (*p* = 0.03; 400 % increase compared to control).

### Sodium Orthovanadate Does Not Affect mRNA Expression of COX-1 and COX-2 Enzymes

Sodium orthovanadate has no statistically significant effect on the mRNA levels of COX-1 and COX-2 in THP-1 cells incubated with or without NS-398.

## Discussion

### The Effect of Sodium Orthovanadate on the Activity of COX-1 and COX-2

At concentrations used in the tests, the studied vanadium compound increases the quantities of PGE_2_ released into the medium by macrophages. This effect is dependent on vanadium concentration. The addition of a specific COX-2 inhibitor, NS-398, leads to a minor reduction in the amount of PGE_2_ being released. Sodium orthovanadate, as an inhibitor of PTP, may increase the activity of COX-2 by phosphorylating tyrosine residues within its molecule against COX-1 protein [17]. However, no change in the growing trend in PGE_2_ release was observed in our studies upon the use of a COX-2 inhibitor. This shows that sodium orthovanadate at concentrations used has no effect on the activity of COX-2.

The increased PGE_2_ release observed in our studies may be due to an increase in the activity of cPLA_2_. cPLA_2_ acts as another, besides COX-2, checkpoint in the synthesis of PGE_2_. By its activity, cPLA_2_ regulates the supply of arachidonic acid for the entire cyclooxygenase and lipoxygenase synthetic pathway. This hypothesis may find its confirmation in studies conducted in leukemia U-937 and BL-60 cell lines, revealing significant increase in the release of arachidonic acid in presence sodium vanadate at concentration of 20 μM [18].

In our studies, sodium orthovanadate had no effect on the synthesis of TXB_2_ while the use of the inhibitor caused a statistically insignificant increase in the level of the synthesized compound. Probably, the inhibitor increased the availability of arachidonic acid for COX-1 molecules by inhibiting the activity of COX-2. The obtained results also suggest that COX-1 is the main enzyme responsible for thromboxane synthesis.

### The Effect of Sodium Orthovanadate on the Expression of COX-1 and COX-2 Proteins

To date, few studies have been conducted on the effect of vanadium compounds on the expression of COX-1. In our studies, we were the first to demonstrate a ca. 10 %, significant reduction in COX-1 expression by micromolar concentrations of sodium orthovanadate. COX-1 is a constitutive enzyme containing an Sp1-binding motif within the *PGHS-1* promoter and activator protein 1 (AP-1)-binding motif within intron 8 [19]. Nonetheless, it was demonstrated that at low concentrations, sodium orthovanadate might, by its effect on AP-1 and Sp1, alter the expression of COX-1, by activating the nuclear factor erythroid 2-related factor-2 (Nrf2), within ERK MAPK pathway [20]. Nrf2 is a transcription factor responsible for the expression of antioxidative and phase II detoxicative enzymes. The expression of these enzymes leads to reduced cytoplasmic levels of ROS, thus inhibition activation of JNK MAPK [21]. This may disturb the activity of AP-1 and cause Sp1 degradation [22]. This in turn cancels out the effect of ROS on increased COX-1 expression. At higher concentrations, sodium orthovanadate activates JNK MAPK and AP-1 [23, 24]. These processes cancel out the effect of vanadium compounds on Nrf2. It is probably for this reason that 10 μM sodium orthovanadate causes no reduction in COX-1 expression.

In our studies, sodium orthovanadate in the presence of the inhibitor did not cause to changes in COX-1 protein expression. This might be due to the levels of 15-deoxy-Δ^12,14^-prostaglandin J_2_ (15d-PGJ_2_) being reduced by the COX-2 inhibitor, thus leading to increased activation of AP-1 [25, 26]. This process canceled out the effect of increased expression of antioxidative enzymes.

Studies conducted by Hirai et al., (1997) in human umbilical vein endothelial cells (HUVEC) showed that sodium orthovanadate had no effect on COX-1 protein expression while simultaneously increasing the COX-1 mRNA levels [27]. However, the tested vanadium compound in the cited study caused such effect in an exposure time-dependent manner.

In order to better understand the effect of sodium orthovanadate in short-term (several days long) anticancer therapy and long-term antidiabetic therapy, one should thoroughly examine the effect of this compound on the expression of COX-1 protein depending on the exposure time.

In our studies, sodium orthovanadate had no significant effect on the expression of COX-2 protein within macrophages cultured without the addition of the inhibitor. However, studies conducted in non-small-cell lung carcinoma A549 cells [27] and HUVEC cells [24] revealed an increase in COX-2 protein expression, although sodium orthovanadate levels used in these studies were much higher than those used in our study [24, 27].

The use of a COX-2 inhibitor caused a significant increase in COX-2 protein expression in our study. Regulation of COX-2 protein expression is a complex process involving positive and negative feedback loops [28]. The expression of COX-2 is inhibited in an autocrine manner by 15d-PGJ_2_ [28]. Gilroy et al., (1999) and Inoue et al., (2000) demonstrated that the use of a specific COX-2 inhibitor leads to inhibition in the activity of COX-2 and thus to reduction in 15d-PGJ_2_ levels [25, 28]. 15d-PGJ_2_ disturbs activation of NF-κB and its ability to bind DNA [29]. Reduced levels of this prostanoid lead to inactivation of the negative feedback loop acting on NF-κB, AP-1, and cAMP response element-binding protein (CREB), resulting in increased COX-2 expression [28–30]. These processes may occur in a peroxisome proliferator-activated receptor-γ (PPARγ)-dependent manner, as this receptor is present in macrophages of the THP-1 cell line [31]. Activated PPARγ disturbs activation of AP-1 by JNK MAPK and binds the cAMP response element (CBP) [26]. Activation of NF-κB caused by reduced 15d-PGJ_2_ levels may be intensified by the studied vanadium compound, as it was demonstrated to activate NF-κB already at the levels of 20 μM [32].

According to Murakami et al. (1997), regulation of COX-2 protein expression involves also a positive feedback loop [33]. PGE_2_, acting via its receptors prostaglandin E_2_ receptor-2 (EP_2_) and prostaglandin E_2_ receptor-4 (EP_4_), acts in an autocrine manner to increase cytoplasmic cAMP levels and thus COX-2 protein expression [28, 33]. Increased cAMP levels activate protein kinase A (PKA) which, by phosphorylating CREB protein leads to an increased COX-2 expression [34]. CREB may be additionally activated by the transduction of signal onto PI3K by activated EP_4_ receptor [35]. The same receptor may also increase the stability of COX-2 mRNA by activating p38 MAPK [36, 37].

Addition of a COX-2 inhibitor, by reducing the quantities of PGE_2_ being released, reduces the cellular cAMP levels thus reducing COX-2 protein expression. At the same time, sodium orthovanadate, by inhibition of the activity of PTP, affects activation of MAPK cascades, which in turn increase COX-2 expression by means of AP-1 and CREB activation [38].

Combination of all of the above processes may lead to increased expression of COX-2 protein in cells cultured with a COX-2 inhibitor and sodium orthovanadate, as observed in our study. This may be of importance in combination experimental antidiabetic and anticancer therapy with the use of vanadium and non-steroidal anti-inflammatory drugs (NSAID), leading to increased COX-2 expression and enhancement of inflammatory processes.

### The Effect of Sodium Orthovanadate on the mRNA levels of COX-1 and COX-2

The studied vanadium compound had no effect on the mRNA levels of COX-1 and COX-2; however, in the studies by Hirai et al. (1997), 10 μM sodium orthovanadate caused an increase in the COX-1 and COX-2 mRNA depending on the HUVEC cell line incubation times [27]. The authors of the cited study observed rapid increase in mRNA levels, reaching a peak value after several hours and then returning to the baseline values [27], which might explain the lack of changes observed in the mRNA levels of COX-1 and COX-2 following 48 h of incubation with sodium orthovanadate.

### Medical Implications of the Obtained Results

The increase in the levels of PGE_2_ induced by sodium orthovanadate treatment was observed. In addition, orthovanadate combined with COX-2 inhibitor was shown to increase the expression of COX-2. Due to the fact that the increased activity of COX-2 promotes tumor growth, NSAID may therefore be recommended in adjuvant anticancer therapy [39, 40]. Despite the fact that increased expression of COX-2 upon the use of the inhibitor does not lead to an increase in the activity of this enzyme, sodium orthovanadate combined with NSAID may trigger cellular signaling pathways that lead to the synthesis of other proinflammatory compounds.

Understanding the mechanisms of vanadium compounds would permit to reduce the side effects of treatments being developed or question the use of the studied vanadium compounds in therapy.

## Abbreviations

15d-PGJ_2_: 15-deoxy-Δ^12,14^-prostaglandin J_2_
AP-1: Activator protein 1
BMOV: Bis(maltolato)oxovanadium(IV)
CBP: cAMP response element
CREB: cAMP response element-binding protein
COX-1: Cyclooxygenase-1
COX-2: Cyclooxygenase-2
cPLA_2_: Cytosolic phospholipase A_2_
EP_2_: Prostaglandin E_2_ receptor-2
EP_4_: Prostaglandin E_2_ receptor-4
GLUT-4: Glucose transporter type 4
HUVEC: Human umbilical vein endothelial cells
Na_3_VO_4_: Sodium orthovanadate
Nrf2: Nuclear factor erythroid 2-related factor-2
NSAID: Nonsteroidal anti-inflammatory drugs
PGE_2_: Prostaglandin E_2_
PKA: Protein kinase A
PMA: Phorbol myristate acetate
PPARγ: Peroxisome proliferator-activated receptor-γ
PTP: Protein tyrosine phosphatase
qRT-PCR: Quantitative real-time PCR analysis
TXA_2_: Thromboxane A_2_
TXB_2_: Thromboxane B_2_
